# Pluripotency and cellular differentiation miRNAs expressed in human blastocoel fluid are associated with embryo quality

**DOI:** 10.1007/s10815-026-03861-x

**Published:** 2026-04-09

**Authors:** Rebeca González-Fernández, Andrea Martínez-López, Jairo Hernández, Pablo Martín-Vasallo, Angela Palumbo, Julio Ávila

**Affiliations:** 1 Laboratorio de Biología del Desarrollo, UD de Bioquímica y Biología Molecular, Universidad de La Laguna, San Cristóbal de La Laguna, Spain; 2 Instituto de Tecnologías Biomédicas, Universidad de La Laguna, San Cristóbal de La Laguna, Spain; 3 Centro de Asistencia a la Reproducción Humana de Canarias, San Cristóbal de La Laguna, Spain

**Keywords:** miRNA, Blastocoel fluid, Embryo quality, Embryo transfer, Pluripotency, Cellular differentiation

## Abstract

**Purpose:**

To analyse the microRNAs (miRNAs) present in blastocoel fluid (BF) and assess the relationship with blastocyst quality.

**Methods:**

Seventy-two blastocysts from 57 women (25 IVF patients and 32 oocyte donors) undergoing IVF in a single private Fertility Center were included. Blastocyst quality was evaluated using Gardner´s classification. BF was collected prior to cryopreservation. MiRNA sequencing (miRNA-Seq) was employed to analyse pooled blastocoel fluid from 8 good-quality (GQ) and 8 medium/poor-quality embryos (PQ). Differential expression of miRNAs was validated in individual BF by relative quantitative real-time PCR (qRT-PCR) in 18 good-quality and 38 medium/poor-quality embryos. Target genes were identified using in silico prediction algorithms.

**Results:**

Using miRNA-seq we identified 85 mature and 75 immature miRNAs and selected 16 miRNAs differentially expressed in GQ and PQ embryos, with a read count exceeding 10. At the p < 0.05 level, eight miRNAs were upregulated in GQ compared to PQ embryos (miR-7107-5p, miR-4687-3p, miR-6743-5p, miR-4651, miR-6503, miR-4516, miR-4472.2, miR-409) and 8 were upregulated in PQ compared to GQ embryos (miR-6805-3p, miR-663a, miR-4438, miR-6068, miR-1182, miR-2682, miR-1908, miR-4754). In individual BF, qRT-PCR identified significantly lower expression of miR-663a, miR-7107-5p and miR-4687-3p in GQ embryos. Hatched GQ embryos had lower expression of miR-6805-3p, miR-663a and miR-4651 compared to unhatched GQ embryos. In silico predicted target genes were associated with cellular growth and differentiation, apoptosis, pluripotency, and embryonic development.

**Conclusion:**

Specific expression profiles of BF microRNAs involved in pluripotency and cellular differentiation may be associated with blastocyst quality and hatching and represent potential biomarkers for successful embryo transfer.

**Supplementary Information:**

The online version contains supplementary material available at 10.1007/s10815-026-03861-x.

## Introduction

Single embryo transfer is a global trend in "in vitro" fertilization (IVF) to reduce the risk of multiple births and pregnancy complications. Therefore, methods for selecting embryos with the greatest potential for development and implantation are critical to maximizing the probability of a successful pregnancy [1, 2]. Embryos are usually evaluated morphologically using Gardner’s morphological classification [3] however this does not fully reflect the processes occurring in the embryo [4].

MicroRNAs (miRNAs) are small noncoding RNAs (18–22 nucleotides in length) that modulate gene expression by regulating essential transcriptional processes including growth, differentiation, and apoptosis [5, 6]. miRNAs are found in the intracellular environment, but they can also be secreted by cells and act as signalling molecules. In the context of human reproduction, miRNAs have been detected in amniotic fluid, blood, semen, follicular fluid, and embryonic cells [7–11]. miRNAs have been proposed to play a role in folliculogenesis, oocyte maturation, early embryonic development, and implantation [12–16]. For example, miR-92 and miR-130b are widely expressed in follicular fluid from oocytes that failed to be fertilized [17]. In addition, it has been observed that some miRNAs are differentially expressed in cumulus cells from immature and mature oocytes, showing a possible role in oocyte maturation [18]. Notably, miR-21-5p is significantly overexpressed in cumulus cells of women with poor ovarian response (POR) [19]. After fertilization embryonic cells differentiate into the inner cell mass and trophectoderm linage coincident with cavitation. On day 4 of human development the embryo secretes fluid to generate blastocoel fluid (BF). Human BF has been shown to include miRNAs that reflect the miRNA profile of embryonic cells. These miRNAs are critical regulators of early embryonic development [20].

miR-106a, miR-17, miR-19b, miR-20 and miR-92a are highly expressed in the human blastocyst and are critical to stem cell differentiation [21, 22]. A recent study by Battaglia et al. found that six specific miRNAs present in human BF (miR-106a, miR-136, miR-203, miR-367, miR-373, and miR-520d) are significantly upregulated in blastocysts that successfully implant, suggesting their potential utility as biomarkers of embryo selection for transfer [20]. On the other hand, prior studies showed that the expression profiles of BF-derived miRNAs may also reflect chromosomal abnormalities in the embryo [23]; for instance, Esmaeilivand et al. reported that levels of miR-20a, miR-661 and miR-142 were significantly different between euploid and aneuploid blastocysts [24]. Since the profile of BF miRNAs varies with blastocyst quality, these miRNAs could be used to determine embryonic potential in IVF cycles. The purpose of the present study was to analyse the miRNAs present in BF to seek relationships with blastocyst quality.

## Material and methods

### Embryos and patients

A total of 72 embryos from 32 egg donors and 25 patients with no ovarian pathology (male or tubal factor infertility) undergoing IVF in a single private Fertility Center from June 2020 to June 2024 were included. This project was approved by the Ethics Committee of the Universidad de La Laguna (CHUC_2018_694.2, 2018/21/01). Ovarian stimulation was carried out with an antagonist protocol using a combination of recombinant FSH and purified urinary gonadotropins. Final oocyte maturation was triggered with a Gonadotropin Releasing Hormone agonist (GnRHa) (decapeptyl 2 mg, IPSEN, Spain) in all egg donors and with a combination of GnRHa and urinary hCG (Gonasi 5000, IBSA, Spain) in IVF patients. Ultrasound-guided egg retrieval was performed 35½ hours later. In vitro fertilization was performed by ICSI (Intracytoplasmic Sperm Injection) and the embryos were grouped according to their quality following Gardner’s classification [3].

### Embryo culture and sample collection

All embryos were cultured in a time-lapse incubator (GERI, Genea, Australia). CSCM-NX sequential culture medium (Irvine scientific, USA) was used for embryo culture. The blastocyst was manipulated in G-MOPS Plus culture medium (Vitrolife, Sweden) under micromanipulation at 400 × magnification on day 5 or 6 of embryo culture. The blastocoel fluid was collected with an ICSI pipette (Vitrolife, Sweden) and the fluid obtained was deposited in a 2 μL drop of RNAse-free buffer (Tris 10 mM and EDTA 1 mM) for further analysis. Immediately after obtaining the blastocoel fluid, the embryos were vitrified with Vitrification freeze Kit (Fujifilm, Netherlands) according to the manufacturer’s instructions.

### Blastocoel fluid RNA isolation

Total RNA, including cell-free circulating and exosomal miRNA, was isolated from blastocoel fluid using the Plasma/Serum RNA Purification Mini Kit (Norgen Biotek, Canada), appropriate for low concentrations and small sample volumes. Initially, samples were brought up to 200 μL using Nuclease-free water and then processed according to the manufacturer’s instructions. Briefly, a lysis buffer was added to obtain the lysis of exosomes and passed through a silica column. The RNA bound to the column was treated with DNase for maximum removal of residual DNA using Norgen’s RNase-Free DNase I Kit (Norgen Biotek, Canada). After an additional wash step, the RNA was eluted in 20 µL of elution buffer.

### miRNA quantification

miRNA concentration was quantified using the Qubit® microRNA Assay Kit, which allows detection from 25 pg/μL to 150 ng/μL, in a Qubit4 Fluorometer (Invitrogen), according to the manufacturer’s instructions at the Genomics Service of SEGAI (Servicio General de Apoyo a la Investigación) of La Laguna University.

Blastocoel fluid miRNA was quantified directly in raw blastocoel fluid as well as after performing an RNA extraction procedure that includes a lysis step. Briefly, the standards were prepared by diluting 10 μL of Standard 1 and 10 μL of Standard 2 in working solution to a final volume of 200 μL. After thorough mixing, the instrument was calibrated with both standards. Fluorescence was measured in the test samples using the total volume obtained for the measurement (approximately 2 μL of raw blastocoel fluid or 20 μL of purified miRNA isolated from blastocoel fluid using the Plasma/Serum RNA Purification Mini Kit), brought to a final volume of 200 μL. Quantification was performed in three independent replicates of raw blastocoel fluid and four independent replicas of purified miRNA).

### miRNA next-generation sequencing (NGS)

Two pools of blastocoel fluid obtained from 8 good quality (GQ) and 8 medium/poor quality embryos (PQ) were sequenced. Library preparation and massive miRNA sequencing were performed by the Nucleus Sequencing Service (University of Salamanca, Spain) using the SMART ultra-low input smRNA-Seq protocol in an Illumina platform, yielding a total of 12,310,287 reads. Data analysis from the sequencing was performed by the Bioinformatics Service of Nucleus, University of Salamanca. Briefly, sequencing quality was assessed using the FASTQC software. The Nextflow’s smRNA-seq protocol was used to read alignment and for quantification against reference sequences. The alignment was performed against the miRBase (version 22.1). Differential expression analysis of miRNA in the two groups was performed using the EdgeR software.

### cDNA synthesis and qRT-PCR

Differential expression of miRNAs was validated in individual BF by relative quantitative real-time PCR (qRT-PCR) in 18 good-quality and 38 medium/poor-quality embryos. Reverse transcription of miRNA was performed from raw blastocoel fluid or after previous RNA isolation using the microScript microRNA cDNA Synthesis Kit (Norgen Biotek,Canada), following the manufacturer’s instructions. Poly A tail was first added to the RNA template and an adapter primer was used for cDNA synthesis. Individual BF samples from 18 GQ and 38 PQ were analysed. qRT-PCR was performed using 2 × Sso Fast Eva Green Supermix (Bio-Rad Laboratories, Hercules, California) in a 10 µL final volume containing 2 × Sso Fast Eva Green Supermix (100 mmol/L KCl, 40 mmol/L Tris–HCl pH 8.4, 0.4 mmol/L of each nucleoside triphosphate, iTaq DNA polymerase 50 U/mL, 6 mmol/L MgCl2, SYBR Green I, 20 nmol/L fluorescein, and stabilizers (Bio-Rad Laboratories, Hercules, California) and 0.25 µM of each forward primer (Supplemental Table 1). miRNA previously detected in blastocoele fluid (miR-92a, miR-143, Let7b and let7i) [25], were used as positive control to test the detection and quantification methodology (Supplemental Table 1). Reactions were carried out in duplicate into a Bio-Rad CFX96 real-time PCR system (Bio-Rad Laboratories, Hercules, California). The qPCR conditions included an initial denaturation at 95 °C for 30 s, followed by 45 cycles performed at 95 °C for 5 s and 60 °C for 5 s. Finally, a melting curve program at 65 °C to 95 °C was done to validate the specificity of the PCR product. Relative expression levels of studied miRNA were normalized using the synthetic RNA cel-miR-39 (Norgen Biotek, Canada). A regression curve was generated from serial dilutions of the synthetic miRNA, and the resulting amplification curve was used to calculate miRNA quantities according to the equation: Quantity = 10^(Ct − b)/m, where b represents the x-intercept and m the slope of the regression curve. To identify and exclude outliers of Ct values from the final RT-qPCR statistical boxplot analysis was performed with SPSS 23 software (IBM Corporation, Somers, NY, USA). For this reason, the results do not include these anomalous values, and the number of embryos included varied among the different miRNAs analysed. Final sample size, indicated in each figure, does not include these outlier values.

### Statistical analysis

Statistical analysis was performed with SPSS 23 software (IBM Corporation, Somers, NY, USA) using the U-Mann Whitney test to evaluate the differences between groups. Groups were initially defined according to embryo quality independent of the hatching process. At a second step we also analysed miRNA expression as a function of the hatching process in both GQ and PQ embryos. A *p* < 0.05 was considered significant.

### In silico analysis

Target genes were identified using in silico prediction algorithms. miRPath (http://www.microrna.gr/miRPathv3) was used for the target gene enrichment of Gene Ontology (GO, https://geneontology.org/docs/go-enrichment-analysis/) and KEGG (Kyoto Encyclopedia of Genes and Genomes, https://www.genome.jp/kegg/). This database uses experimentally validated miRNA-gene interactions (miRTarbase, Diana-LncBase) or sets of predicted miRNA targets (TargetScan, Diana-microT-CDS). Significant results for pathway analyses are adjusted for FDR (false discovery rate) (*p* < 0.05).

## Results

### miRNA detection and quantification

miRNA detection and quantification by Qubit as well as qRT-PCR amplification of control miRNAs were only possible after RNA isolation using the Plasma/Serum RNA Purification Mini Kit, whereas direct analysis of blastocoel fluid without prior extraction yielded no detectable signal. To have a reference value, using Qubit, we measured miRNA concentration in four individual blastocoel fluids obtaining a value of 0.12 ± 0.03 ng/μL.

### miRNA sequencing

Using miRNA-seq analysis, we identified 85 mature- and 75 immature miRNAs. The quantification of both immature miRNA (precursor) and mature miRNA levels is important because a disruption at any stage of the miRNA biogenesis pathway can lead to disease-associated alterations. We selected 16 miRNAs that were differentially expressed in good and medium/poor-quality embryos with a read count exceeding 10 (Table 1). Eight miRNAs were significantly upregulated in good quality compared to medium/poor quality embryos (miR-7107-5p, *p* = 0.022; miR-4687-3p, *p* = 0.033; miR-6743-5p, *p* = 0.036 miR-4651, *p* = 0.045; miR-6503, *p* = 0.018; miR-4516, *p* = 0.026; miR-4472.2, *p* = 0.026; miR-409, *p* = 0.032) and eight miRNAs displayed significantly higher expression in medium/poor quality than in good quality embryos (miR-6805-3p, *p* = 0.001; miR-663a, *p* = 0.002; miR-4438, *p* = 0.001; miR-6068, *p* = 0.0005; miR-1182, *p* = 0.008; miR-2682, *p* = 0.008; miR-1908, *p* = 0.013; miR-4754, *p* = 0.03).

**Table 1:** Differentially expressed miRNAs identified by miRNA-seq in embryos of good quality (GQ) and medium/poor quality (PQ)

a) Mature miRNAs			
*miRNA*	*Counts*		*P Value*
	GQ	PQ	
*miR-6805-3p*	14.5	124	0.001
*miR-663a*	36	260	0.002
*miR-7107-5p*	1460	124	0.022
*miR-4687-3p*	3209.5	313	0.033
*miR-6743-5p*	428	44	0.036
*miR-4651*	906.5	103	0.045
b) Immature miRNA			
*miRNA*	*Counts*		*P Value*
	GQ	PQ	
*miR-6068*	10.5	111	0.0005
*miR-4438*	10	85	0.001
*miR-1182*	25	130	0.008
*miR-2682*	16.5	92	0.008
*miR-1908*	70	331	0.013
*miR-6503*	425	35	0.018
*miR-4516*	2752	265	0.026
*miR-4472.2*	2609	251	0.026
*miR-4754*	58	215	0.03
*miR-409*	512	49	0.032

### qRT‐PCR miRNA expression in blastocoel fluid according to blastocyst quality

**Fig. 1:**
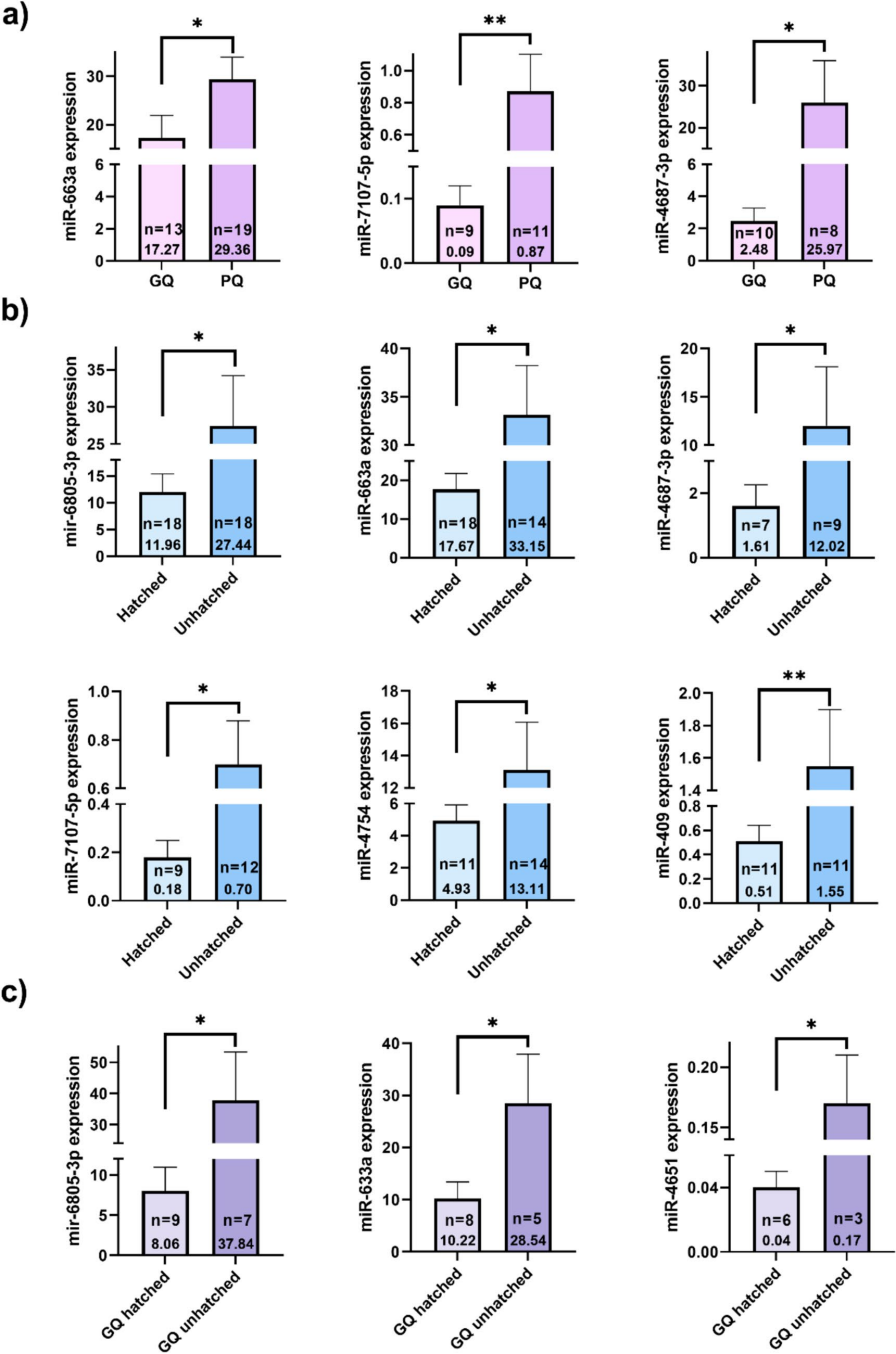
Histogram representation of: **a** miR-663a, miR-7107-5p and miR-4687-3p expression levels in morphologically good-quality and medium/poor-quality embryos; **b** miR-6805-3p, miR-663a, miR-4687-3p, miR-7107-5p, miR-4754 and miR-409 expression levels in hatched and unhatched embryos; **c** miR-6805-3p, miR-663a and miR-4651 expression levels in hatched and unhatched good-quality embryos (GQ). Data are shown as mean ± SD (*) = *p* < 0.05; (**) = *p* < 0.01. GQ = good quality; PQ = medium/poor quality embryos

RT-qPCR was used to confirm differential miRNA expression in individual BF for good-quality (*n* = 18) and medium/poor-quality embryos (*n* = 38). Expression of miR-2682 and miR-6503 was too low to be detected in most of the cases, so its relationship with morphological embryo parameters could not be assessed. Comparison of miRNA expression between good- and medium/poor-quality embryos shows that miR-663a, miR-7107-5p and miR-4687-3p expression was lower in good quality embryos (*p* = 0.048, *p* = 0.002 and *p* = 0.021, respectively) (Fig. 1a). Regarding the hatching process, independent of quality grading, hatched embryos showed lower expression of some mature miRNAs (miR-6805-3p, *p* = 0.021; miR-663a, *p* = 0.015; miR-7107-5p, *p* = 0.025 and miR-4687-3p, *p* = 0.020) and some immature miRNAs (miR-4754, *p* = 0.029 and miR-409, *p* = 0.006) (Fig. 1b).

Furthermore, when we combined both variables, quality and hatching, we detected that in high quality embryos, expression levels of miR-6805-3p (*p* = 0.047), miR-663a (*p* = 0.049) and miR-4651 (*p* = 0.012) were lower in hatched vs unhatched blastocysts. In contrast, in medium/poor quality embryos regardless of the hatching state there was no difference in the miRNA expression (Fig. 1c).

### In‐silico pathway analysis

**Fig. 2:**
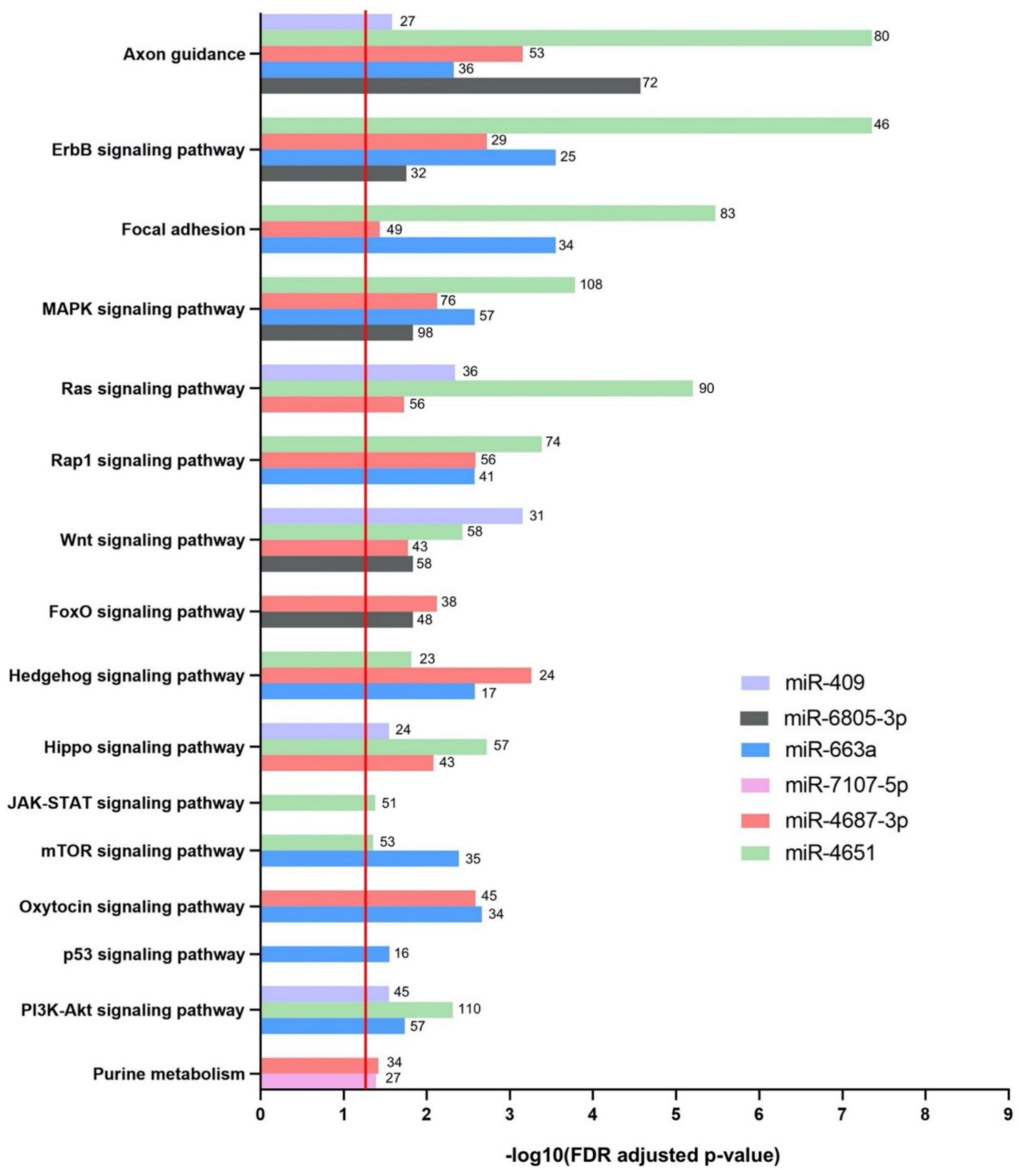
Pathway enrichment analysis of BF-miRNAs using miRPath v3.0. Coloured bars indicate KEGG pathways significantly enriched for predicted targets of individual BF-miRNAs: green for miR-4651, red for miR-4687-3p, blue for miR-663a, pink for miR-7107-5p, grey for miR-6805-3p, and purple for miR-409. The red horizontal line denotes the threshold for statistical significance (*p* < 0.05). Numbers within each bar represent the total number of genes associated with the respective pathway

KEEG Pathway analysis of differentially expressed miRNAs, (miR-6805-3p, miR-663a, miR-7107-5p, miR-4687-3p, miR-4651, miR-4754 and miR-409) was performed. Among the various signalling pathways and processes, we focused on those associated with biological mechanisms implicated in embryonic development. These included those involved in cell adhesion, morphogenesis and signalling cascades such as axon guidance, Hippo, p53, Wnt, PI3K-Akt, FoxO, ErbB, MAPK, Hedgehog, mTOR, Rap1, and Jak-STAT signalling, as well as focal adhesion and purine metabolism (Fig. 2).

## Discussion

This study provides new insights into the contributions of blastocoel fluid miRNAs during human embryonic development, and their potential as non-invasive markers of embryonic quality. Our findings confirm that blastocoel fluid miRNAs are predominantly contained within exosomes, as they are not detectable without an extraction step that includes exosome lysis. This supports the hypothesis that miRNAs contained in blastocoel fluid do not represent cellular waste, but rather a specific and regulated message mediated by exosome trafficking [26].

To identify miRNAs of interest in embryonic development, we performed NGS on pooled blastocoel fluid from morphologically high-quality versus medium/poor-quality embryos and found 85 mature and 75 immature miRNAs whose read counts differed significantly between the two conditions. Mature miRNAs are the functional effectors that execute the regulatory role, but the level of immature miRNAs provides vital clues about the underlying transcriptional activity, processing efficiency, and stability. Quantifying both immature and mature miRNA levels is critical, as disruptions at any stage of the miRNA biogenesis pathway can result in disease-associated alterations, as has been demonstrated in chronic prostatitis, ischemic cardiomyopathy, and multiple cancer types [27–30]. The present results support the hypothesis that miRNAs present in BF may modulate gene expression and play a role in the molecular mechanisms and signalling involved in the early stages of embryonic development.

Among the differentially expressed miRNAs, those with sufficient copy numbers were selected for further analysis by qRT-PCR in blastocoel fluid of individual embryos. In agreement with a recent study by Battaglia et al. [31] that reported overexpression of 11 miRNAs in implanted high-quality embryos, our miRNA-seq results reveal that 6 of these miRNAs (miR-520b, miR-520d, miR-202a, miR-373, miR-525-3p and miR-532-3p) are overexpressed in BF from good-quality embryos (data not shown). However, they did not present enough copies for subsequent analysis by qRT-PCR.

miR-663a, miR-7107-5p, and miR-4687-3p were expressed at lower levels in high-quality embryos compared to medium/poor quality embryos (Table 2). It is known that miRNAs can control the fate of embryonic pluripotent cells by directly targeting the 3’ UTR or binding to the coding sequence of pluripotency factors, modulating their expression [32]. Based on this, the high expression of these miRNAs in medium/poor-quality embryos may indicate a decrease in the pluripotent state, potentially repressing transcription factors or signalling regulators critical for maintaining the self-renewing state of the inner cellular mass (ICM).

**Table 2:** This table represents the statistical significance in the differential expression of the selected miRNA in individual embryos of good vs. poor quality blastocysts (GQ/PQ), hatched vs. unhatched blastocysts, GQ Hatched vs. GQ Unhatched blastocysts and PQ Hatched vs PQ Unhatched blastocysts

*miRNA*	*GQ/PQ*	*Hatched/Unhatched*	*GQ Hatched/GQ Unhatched*	*PQ Hatched/PQ Unhatched*
*miR-663a*	*P* < 0.05	*P* < 0.05	*P* < 0.05	NS
*miR-7107-5p*	*P* < 0.01	*P* < 0.05	NS	NS
*miR-4687-3p*	*P* < 0.05	*P* < 0.05	NS	NS
*miR-6805-3p*	NS	*P* < 0.05	*P* < 0.05	NS
*miR-4754*	NS	*P* < 0.05	NS	NS
*miR-409*	NS	*P* < 0.01	NS	NS
*miR-4651*	NS	NS	*P* < 0.05	NS

**Fig. 3:**
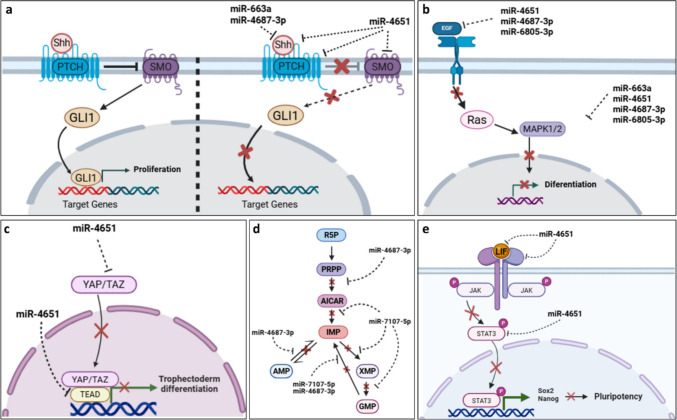
Functional implications of miRNAs in key signalling pathways of early embryonic development. **a** Hedgehog signalling. **b** EGF-Ras-MAPK signalling. **c** Hippo signalling. **d** Purine biosynthesis and metabolism. **e** JAK-STAT signalling. Dashed lines represent predicted regulatory interactions

The functional implications of miR-663a and miR-4687-3p have been reported in various oncological models and stem cell lines, where they enhanced cellular proliferation, migration, and invasion [33–35]. In addition, the in-silico analysis shows that both miRNAs play a role in regulating Sonic Hedgehog (SHH) pathway, which has been linked to developmental competence and IVF embryo quality in a porcine model [36]. Elevated expression of these miRNAs in low-quality embryos could interfere with SHH signalling by downregulating essential genes such as SHH, GLI, or SMO, ultimately impairing the regulation of cellular migration and proliferation during embryogenesis (Fig. 3a).

The functional role(s) of miR-7107-5p remain poorly defined, but it showed similar correlations and targets as miR-4687-3p in pathways such as purine metabolism, essential for nucleotide biosynthesis, DNA replication and RNA transcription (Fig. 3d). Notably, during the preimplantation period, de novo purine synthesis is essential to support rapid cell proliferation and the activation of the embryonic genome, as shown in mouse models [37, 38]. Therefore, dysregulation of these miRNAs could negatively affect metabolic readiness and developmental competence at these early stages.

Following Gardner’s classification for ICM, trophectoderm (TE) and degree of expansion, we classified blastocysts as medium-poor quality and good quality, without taking hatching into account. Subsequently, we analysed separately the hatching process and found that hatching blastocysts have lower expression of the same miRNA that have low expression in GQ blastocysts (miR-663a, miR-7107-5p, and miR-4687-3p). Additionally, hatching blastocysts also displayed lower expression of miR-6805-3p, miR-4754 and miR-409 (Table 2).

Recent findings from Russell et al. [14] indicate that miR-409 is enriched in human ICM, where it may help preserve pluripotency by suppressing transcription factors related to cell differentiation. The decrease in miR-409 levels that we have observed in BF may indicate that the mechanisms to maintain pluripotency are loosening, facilitating the initiation of cell differentiation and preparation for implantation. Interestingly, both miR-409 and miR-6805-3p have been implicated in the regulation of apoptotic and angiogenic pathways, such as Hippo, PI3K-Akt and PP2A/p38 [39–41], which supports their potential as modulators of embryonic microenvironment and blastocyst viability. In contrast, miR-4754 did not show statistically significant associations with any specific signalling pathways based on current enrichment analysis.

Moreover, when the hatching process was analysed within the good quality group, we observed reduced expression of miR-6805, miR-663a and miR-4651 in hatching blastocysts. Interestingly, miR-4651 was the only miRNA that did not display differential expression in GQ vs PQ or hatching vs unhatching embryos, but selectively displayed differential expression in hatching vs unhatching good quality embryos (Table 2) This raises the hypothesis that miR-4651 could serve as a valuable biomarker for identifying, within morphologically high-quality embryos, those with a higher implantation and pregnancy potential. This miRNA not only regulates gene expression associated with proliferation and cell cycle (HMGA2, CDK2 and cyclin D) [42], but also regulates key factors of the Wnt, Hippo and Jak-STAT pathways, such as YAP, TEAD2, TAZ and LIF (Fig. 3c-e). All of them are crucial during the first decisions of cellular fate, which lead to the formation of TE and ICM. In particular, the Hippo pathway regulates nucleus exclusion of YAP/TAZ, preventing TEAD activation and transcription of specific target gene of TE differentiation (e.g., CDX2 and GATA3) [43, 44]. By decreasing the availability of these factors, miR-4651 contributes to the preservation of the pluripotent stage. Thus, the dynamic on/off regulation of the activity of the Hippo pathway allows the ICM to preserve its pluripotent stage, while the TE cells move toward differentiation [44–46]. Their higher levels observed in embryos of comparable quality that did not achieve hatching indicate a possible dysregulation between these events, potentially compromising overall embryo quality.

Regarding the pathways described above, in silico analysis showed that multiple miRNAs, including miR-6805-3p, miR-663a, miR-4687-3p, and miR-4651 target key genes in the ErbB signalling pathway (Fig. 3b). This pathway is crucial for proper embryo morphology. In human blastocysts, decreased EGFR expression has been linked to reduced embryo quality following in vitro culture [47]. This supports the hypothesis that excessive regulation by these miRNAs could be blocking crucial pathways for the structural development of the embryo [48].

## Limitations

A limitation of this study is the lack of information on the outcomes of embryo transfers. The sample size is still too small to allow analysis of implantation and pregnancy rates. In addition, studies are ongoing in our laboratory to further investigate the functional validation of these miRNAs on the target genes predicted in silico.

## Conclusions

In this study we demonstrate that blastocoel fluid contains specific exosome encapsulated miRNAs (miR-663a, miR-6805-3p, miR-7107-5p, miR-4687-3p, miR-4754, miR-409, and miR-4651) involved in the modulation of key signalling pathways in early embryonic development, such as Hedgehog, ErbB, MAPK, Wnt, Hippo, purine metabolism and Jak-STAT. Lower expression of certain miRNA (miR-663a, miR-6805-3p and miR-4651) is associated with good embryo quality and successful hatching (improved developmental competency), which suggests that these miRNAs may interfere with cell proliferation, differentiation and pluripotency maintenance and act as negative regulators of embryo competence. Studies are in order to further investigate the basic molecular and signalling processes involved in blastocyst biology. Such research will open new perspectives for the use of blastocoel fluid miRNA composition as a complementary, non-invasive biomarker to enhance the selection of embryos with the highest potential, ultimately improving success rates in assisted reproductive technologies.

## Supplementary Information

Below is the link to the electronic supplementary material.

Supplementary file1 (DOCX 14 KB)

## Data Availability

The data underlying this article will be shared from the corresponding author upon reasonable request.
