# Supplementary material for: Pluripotency and cellular differentiation miRNAs expressed in human blastocoel fluid are associated with embryo quality
Source: J Assist Reprod Genet. 2026 Apr 9;43(6):1785–95. doi: 10.1007/s10815-026-03861-x (PMC13319289; doi:10.1007/s10815-026-03861-x)
Supplement: Supplementary file 1 — Supplementary file1 (DOCX 14 KB) [file 10815_2026_3861_MOESM1_ESM.docx]

**Table 1** Sequence of specific primers used for each microRNA amplification

| miRNA | Sequence (5’-3’) | Tm (ºC) | |
| --- | --- | --- | --- |
| miR-6805-3p | TTGCTCTGCTCCCCCGCC | 69.4 | |
| miR-663a | AGGCGGGGCGCCGCGGGAC | 81 | |
| miR-7107-5p | GGCCTGGGGAGGAGGAAG | 64.4 | |
| miR-4687-3p | TGGCTGTTGGAGGGGGCAG | 69.1 | |
| miR-6743-5p | AAGGGGCAGGGACGGGTG | 67.9 | |
| miR-4651 | GGGGTGGGTGAGGTCGGG | 68.4 | |
| miR-6068 | TTGTGGCTGAGTGTCACGC | 62.2 | |
| miR-4438 | TCTAAGCCTGTGCCTTGCC | 61.5 | |
| miR-1182 | GGGACTTGTCACTGCCTGTC | 60.7 | |
| miR-2682 | TCCAATCTCTTTGGGACGC | 61.1 | |
| miR-1908 | CGGCGATTGGTCCGTATG | 63.4 | |
| miR-6503 | ATCCCCAGAAGCTGAGGATTAG | 60.9 | |
| miR-4516 | CTGTGAGTCAGCCACGGC | 66.7 | |
| miR-4472.2 | TTGAGACAGAGTCTTGCTCCG | 60.7 | |
| miR-4754 | TAGCGGAGTGAGGCCCAG | 62.5 | |
| miR-409 | GGAGAGGTTACCCGAGCAAC | 61.0 | |
| miR-92a | TATTGCACTTGTCCCGGC | 55.6 |  |
| miR-143 | TGAGATGAAGCACTGTAGCTC | 54.3 |  |
| Let7b | TGAGGTAGTAGGTTGTGT | 53.6 |  |
| Let7i | TGAGGTAGTAGTTTGTGCTG | 51.8 |  |

Tm= melting temperature.
